# Trends in Inhaler Prescriptions and Associated Cost in the United States From 2014 to 2018: An Analysis From the Medicare Part D Database

**DOI:** 10.7759/cureus.13498

**Published:** 2021-02-22

**Authors:** Akesh Thomas, Ibrahim Haddad, Girendra Hoskere

**Affiliations:** 1 Internal Medicine, East Tennessee State University Quillen College of Medicine, Johnson City, USA; 2 Pulmonary and Critical Care Medicine, East Tennessee State University Quillen College of Medicine, Johnson City, USA

**Keywords:** copd, health care expenses, inhaler use, medicare part d, asthma

## Abstract

Introduction: Chronic obstructive pulmonary disease (COPD) and asthma constitute the majority of the pulmonary disease burden in the United States. Various kinds of inhalers are used for treating both these conditions, and Medicare is the biggest payer for them. We analyze the trend in prescriptions and associated expenses of various inhaler prescriptions from 2014 to 2018 using the Medicare part D database.

Methods: Medicare part D data is analyzed for the years 2014-2018. Inhalers are grouped based on their drug class. The number of beneficiaries and the associated expenses for each inhaler and the groups were calculated separately and analyzed using statistical software.

Results and Conclusion: Some 85 million beneficiaries received inhalers through Medicare part D over the four years. Medicare spent 50.5 billion US dollars on these prescriptions, which showed an increase of 130% users and 128% expenditure over the four years. Medicare's expense for inhaler prescriptions is growing and is expected to increase even more in the near future.

## Introduction

Chronic obstructive pulmonary disease (COPD) and asthma together constitute the lion's share of pulmonary disease burden in the United States. In 2014-2015, more than six percent of adults in the United States older than 40 years of age reported having a diagnosis of COPD. This is about 0.8% less than that of 2008 and 2009 statistics [1], but still being the third most common cause of mortality affecting around 15.7 million people [2]. As of 2018, 24 million people reported having asthma in the United States. About 19 million are adults over the age of 18 [3]. In the year 2016 alone, asthma was the reason for nine million physician office visits and nearly two million emergency room visits. Medicare covers much of the burden of COPD and asthma. As of 2014, about 4.2% of Medicare beneficiaries reported having asthma [4]. One in nine Medicare beneficiaries was diagnosed with COPD [5]. The Center for Disease Control (CDC) spent 32.1 billion US dollars for COPD in 2010 and was estimated to spend about 49 billion in 2020. Medicare covers 51% of the COPD expense burden. The main expense of COPD and asthma treatment on Medicare falls on part D of Medicare, mostly for buying inhalers. The expense of inhaler prescriptions skyrocketed after the ban of chlorofluorocarbon propellants in 2008, virtually eliminating all the generic inhalers from the market. The changes in the global initiative for chronic obstructive lung disease (GOLD) guidelines in 2017 [6] and the global initiative for asthma (GINA) guidelines in 2019 [7] are estimated to change the type and cost of inhaler prescriptions significantly.

## Materials and methods

A cross-sectional study was done using the Medicare part D prescription drug claims data set from 2014 to 2018 for inhaler prescriptions. A total of 23 inhaler formulations were included. Data were collected and analyzed in September of 2020. Graphs and charts were plotted using Microsoft Excel and R 4.0.2. Inhalers were divided into short-acting beta-agonist (SABA), short-acting muscarinic antagonist (SAMA), inhaled corticosteroids (ICS), long-acting beta-agonist (LABA), long-acting muscarinic antagonist (LAMA), or as different combinations of the above including LABA/LAMA, LABA/ICS, and SABA/SAMA. A table of drugs included in each class is given (Table 1).

**Table 1:** Inhaler drugs under different classes. SABA, short-acting beta-agonist; SAMA, short-acting muscarinic antagonist; ICS, inhaled corticosteroids; LABA, long-acting beta-agonist; LAMA, long-acting muscarinic antagonist

SABA	Albuterol/levalbuterol
SAMA	Ipratropium
ICS	Beclomethasone, fluticasone, mometasone, budesonide, ciclesonide
LABA	Salmeterol, formoterol, arformoterol, indacterol
LAMA	Tiotropium, aclidinium, umeclidinium
LABA/LAMA combination	Tiotropium/oladacterol, umeclidinium/vilanterol, indacaterol/glycopyrium
LABA/ICS combination	Formoterol/mometasone, fluticasone/salmeterol, fluticasone/vilanterol
SABA/SAMA combination	Ipratropium/albuterol

## Results

**Figure 1:**
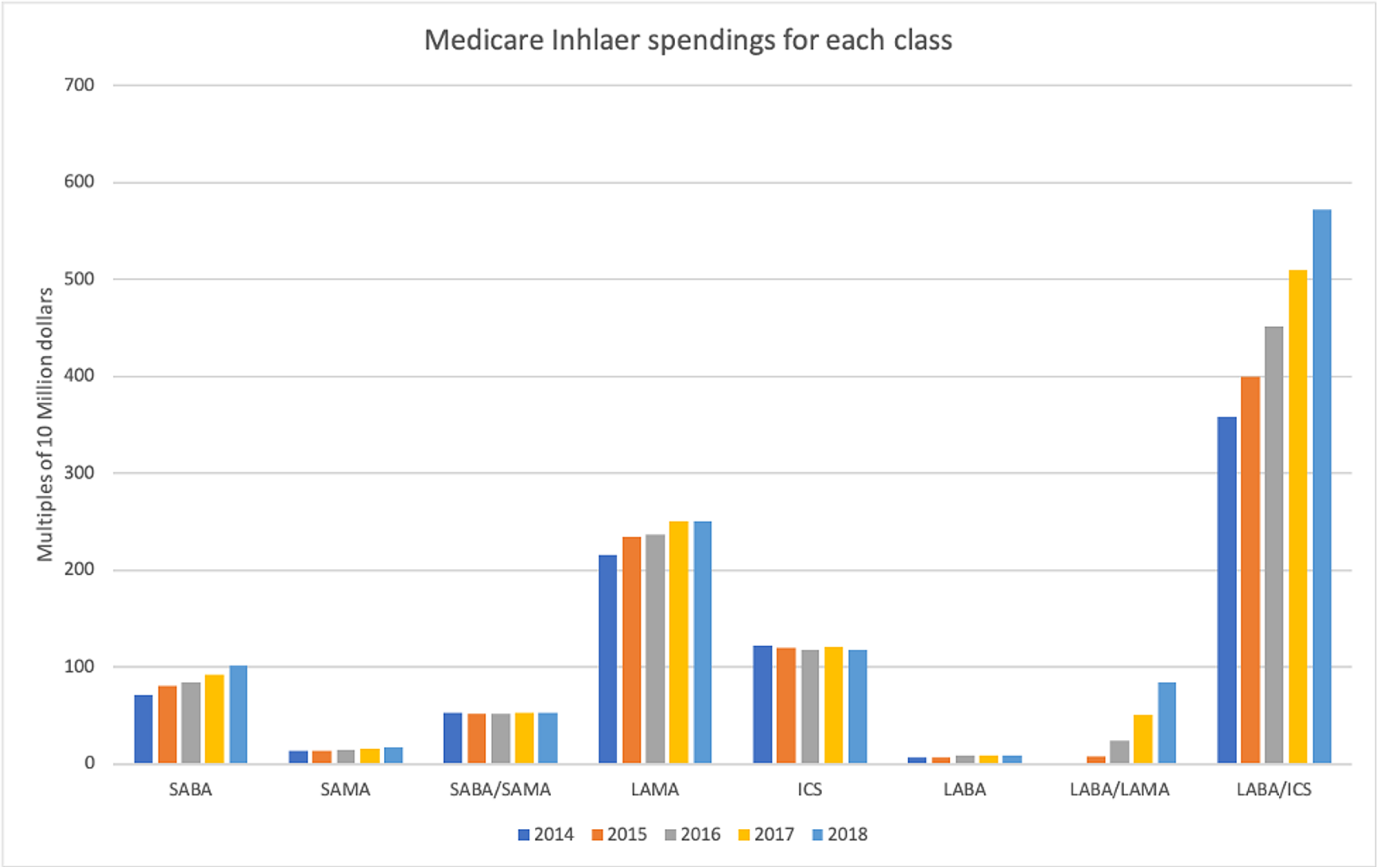
Medicare inhaler spending for each class. SABA, short-acting beta-agonist; SAMA, short-acting muscarinic antagonist; LABA, long-acting beta-agonist; LAMA, long-acting muscarinic antagonist; ICS, inhaled corticosteroid

**Figure 2:**
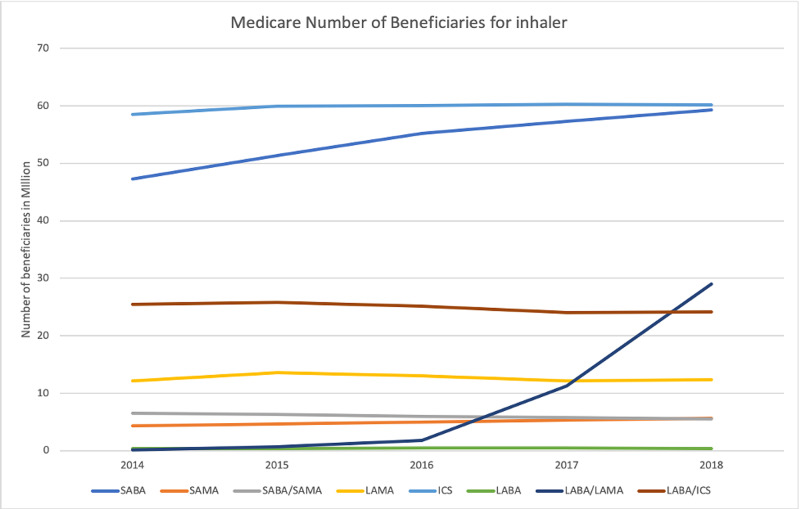
Medicare number of beneficiaries for inhaler classes. SABA, short-acting beta agonist; SAMA, short-acting muscarinic antagonist; LABA, long-acting beta-agonist; LAMA, long-acting muscarinic antagonist; ICS, inhaled corticosteroid

A total of 85,823220 beneficiaries were analyzed. The total number of Medicare part D beneficiaries range from 15,502,524 in 2014 to 19,677,088 in 2018 (127% increase). Medicare spent a total of 50549 million dollars for inhaler prescriptions during the entire study period, ranging from 8422 million in 2014 to 11028 million in 2018 (130% increase). Most amount of money is spent on LABA/ICS combination, and least amount is spent on LABA alone inhalers (Figure 1). Most beneficiaries were for ICS alone prescription, and the least number of prescriptions are for LABA alone prescriptions (Figure 2). The biggest changes in expense and number of beneficiaries are seen in LABA/LAMA combination inhaler, but this will be an unfair comparison since only one preparation of this combination was paid by Medicare in 2014, which increased to three in 2018. Excluding the LABA/LAMA combination, the biggest increase is in SAMA with a 130% increase in the number of beneficiaries and a 128% increase in expenditure. On the other hand, SABA/SAMA combination had the biggest decrease in the number of beneficiaries to 83%, with a cost reduction to 99%.

## Discussion

The number of inhaler prescriptions increased significantly over four years from 2004 to 2008, increasing the total number of beneficiaries by 127%. This can be due to an increase in either asthma or COPD prescriptions. Although the number of people with COPD increased slightly over time, the number of people with asthma increased significantly, especially in the United States [8-10]. This change can be partially attributed to the reduction in tobacco use, which is the significant risk factor for COPD, throughout the nation, and the increase in the prevalence of obesity and air pollution, which are major risk factors for asthma. The expenses calculated in our study are only for the inhaler prescriptions for COPD and asthma; there will be much more expenses to consider if one wants to calculate the total expenditure of Medicare on each disease, including the inpatient expenses, expense for home oxygen, diagnostic expenses, and on other outpatient medications. Other outpatient medications that Medicare is spending for COPD and asthma include medications for refractory COPD like azithromycin, theophylline, and roflumilast, medications for refractory asthma, including the expensive biologics. While comparing the expenses over the years, inflation should also be considered; the total inflation rate from 2014 to 2018 is 5.2% with an average inflation rate of 1.2% [11]. Although nearly 80% of Medicare beneficiaries are over the age of 65, a separate age group-wise analysis was not possible using the available data which is a limitation of the study.

The next expected change is based on the SYGMA trails One and Two published in the New England Journal in 2018, which showed a clear benefit of as needed LABA/ICS combination in maintenance and prevention of severe exacerbations in mild asthma [12-13]. Once incorporated into practice, this change is expected to increase the number of LABA/ICS combination prescriptions and may decrease the number of SABA and ICS prescriptions. This change will further increase the Medicare expenses as the LABA/ICS combinations are more expensive than the others.

## Conclusions

Our study found that the number of beneficiaries and the expenses of Medicare on inhaler prescriptions is steadily increasing and is expected to increase further in the upcoming future. The LABA/LAMA prescriptions showed the highest increase in both the number of users and expense from 2014 to 2018. The next expected increase is in the number of LABA/ICS prescriptions. This will further increase the financial burden on Medicare.
